# Reduction in cardiolipin reduces expression of creatine transporter-1 and creatine transport in growing hCMEC/D3 human brain microvessel endothelial cells

**DOI:** 10.3389/fddev.2023.1158369

**Published:** 2023-03-28

**Authors:** Donald W. Miller, Grant M. Hatch

**Affiliations:** 1 Department of Pharmacology and Therapeutics, University of Manitoba, Winnipeg, MB, Canada,; 2 Children’s Hospital Research Institute of Manitoba, Winnipeg, MB, Canada

**Keywords:** cardiolipin, blood brain barrier, mitochondria, creatine transporter 1, P-glycolprotein, breast cancer resistance protein, drug transport

## Abstract

The phospholipid cardiolipin (CL) regulates mitochondrial energy production. Endothelial cells of the blood-brain barrier (BBB) play a vital role in uptake of metabolites into the brain and are enriched in mitochondria. We examined how deficiency in BBB endothelial cell CL regulates the expression of selected drug and metabolite transporters and their function. Cardiolipin synthase-1 (hCLS1) was knocked down in a human brain microvessel endothelial cell line, hCMEC/D3, and CL levels and the mRNA expression of selected BBB drug and metabolite transporters examined. Mock transfected hCMEC/D3 cells served as controls. Incorporation of (^14^C)creatine and (^14^C)oleate into hCMEC/D3 cells was determined as a measure of solute metabolite transport. In addition, protein expression of the creatine transporter was determined. Knockdown of hCLS1 in hCMEC/D3 reduced CL and the mRNA expression of creatine transporter-1, p-glycoprotein and breast cancer resistance protein compared to controls. In contrast, mRNA expression of ATP binding cassette subfamily C members-1, -3, multidrug resistance-associated protein-4 variants 1, -2, and fatty acid transport protein-1 were unaltered. Although ATP production was unaltered by hCLS1 knockdown, incorporation of (^14^C)creatine into hCMEC/D3 cells was reduced compared to controls. The reduction in (^14^C)creatine incorporation was associated with a reduction in creatine transporter-1 protein expression. In contrast, incorporation of (^14^C)oleic acid into hCMEC/D3 cells and the mRNA expression of fatty acid transport protein-1 was unaltered by knockdown of hCLS1 compared to controls. Thus, knockdown of hCLS1 in hCMEC/D3, with a corresponding reduction in CL, results in alteration in expression of specific solute membrane transporters.

## Introduction

Cardiolipin (CL) is a major polyglycerophospholipid in mammalian tissues and comprises approximately 7%–15% of the entire phospholipid phosphorus mass of mitochondria (Pangborn, 1942; Poorthuis et al., 1976; Hatch, 2004). CL modulates the activity of many mitochondrial enzymes involved in the generation of ATP [reviewed in (Hoch, 1992; Hatch, 2004)] and is the “glue” that holds the mitochondrial electron transport chain together (Zhang et al., 2002; Ascenzi et al., 2011). However, CL has recently been implicated in the regulation of many other cellular processes including apoptosis, autophagy, mitophagy and membrane transport of glucose [reviewed in (Falabella et al., 2021)], (Nguyen et al., 2016; Hsu and Shi, 2017; Choubey et al., 2021). The final step of *de novo* biosynthesis of CL is the conversion of phosphatidylglycerol to CL catalyzed by CL synthase (CLS) localized exclusively to the inner mitochondrial membrane (Hostetler et al., 1971; Schlame and Haldar, 1993). We previously cloned and characterized the murine and human CLS (hCLS1) (Lu et al., 2006).

Barth Syndrome is a rare X-linked genetic disorder, caused by a mutation in the TAFAZZIN gene, and is the only disease in which the specific biochemical defect is a reduction in CL [reviewed in (Barth et al., 2004; Hauff and Hatch, 2006)]. Previous studies have suggested that some Barth Syndrome patients exhibit a cognitive phenotype (Mazzocco et al., 2007). In addition, abnormal CL levels have also been reported in numerous neurodegenerative disorders [reviewed in (Falabella et al., 2021)]. We recently examined how tafazzin deficiency impacts cognitive function in mice (Cole et al., 2018). We showed that tafazzin knockdown in mice resulted in significant memory deficiency based on novel object recognition test. Whether the cognitive defects in tafazzin knockdown mice are related to a loss in CL in blood-brain barrier (BBB) endothelial cells and/or transport of compounds into the brain is unknown. In fact, few studies have examined if altering CL levels regulate uptake and membrane transport processes in mammalian BBB endothelial cells.

In the current study we examined if reduction in CL altered the expression and function of selected BBB endothelial cell transport proteins. We show that knockdown of hCLS1 in hCMEC/D3 human BBB endothelial cells, with a corresponding reduction in CL, results in reduced expression of creatine transporter-1 expression and a reduction in (^14^C) creatine incorporation into these cells. In addition, knockdown of hCLS1 in hCMEC/D3 reduced the mRNA expression of p-glycoprotein and breast cancer resistance protein but not ATP binding cassette subfamily C members-1, -3, multidrug resistance-associated protein-4 variants 1, -2, and fatty acid transport protein-1.

## Materials and methods

### Materials

Cell culture medium (CSC complete medium) and passaging reagents were obtained from Cell Systems Corporation (Kirkland, WA, United States). Human adult brain endothelial cell line hCMEC/D3 was kindly provided by Dr. P.O. Couraud (Institut Cochin, France) in a cryopreserved vial at passage 27. Endothelial basal medium-2 was from Lonza (Basal, Switzerland). Fetal bovine serum (FBS), other medium supplements, cell culturing reagents and primers and reagents used for qPCR were obtained from Life Technologies Inc. (Burlington, ON, Canada) and Sigma-Aldrich (St. Louis, MO, United States). Opti-MEM reduced serum medium, Lipofectamine RNAiMAX Transfection Reagent and the Silencer were obtained from Thermo Fisher (Winnipeg, MB, Canada). Select Pre-designed siRNA for human cardiolipin synthase-1 (CLS) were obtained from Life Technologies Inc. (Burlington, ON, Canada). RNeasy and Plus Mini Kit used for RNA extraction was obtained from Qiagen (Cambridge, MA, United States). Silencer^®^ Select Negative Control No.2 siRNA was from Life Technologies (Burlington, ON). (1–14C)oleate and (^14^C)creatine were obtained from PerkinElmer (Boston, MA, United States). Ecolite scintillant was obtained from ICN Biochemicals (Montreal, QC, Canada). Seahorse XF24 analyzer and reagent kits for determination of the relative contribution of glycolysis and oxidative phosphorylation (OX-PHOS) to basal ATP production were from Agilent (North Billerica, MA, United States). Antibodies to SLC6A8 (Crt1) and cyclophilin were from ThermoFisher (Winnipeg, Canada). HRP-linked monkey anti-rabbit secondary antibody was from GE Healthcare Life Sciences (Little Chalfont, United Kingdom). All other biochemicals were of ACS grade and were obtained from either Sigma-Aldrich or Fisher Scientific (Winnipeg, MB, Canada).

### Transfection of cells

HCMEC/D3 cells were transfected for 48 h with human cardiolipin synthase-1 (hCLS1) siRNA to knock down the CL *de novo* biosynthetic enzyme hCLS1 as previously described (Nguyen et al., 2016). In brief, HCMEC/D3 cells were seeded at a density of 15,000 cells/cm^2^ onto 100 mm dishes in 6 well culture plates. The cells were left to attach for 4–6 h, and the culture medium was then replaced by OptiMEM I reduced serum media. Transfection of subconfluent HCMEC/D3 cells were performed using siRNA against the hCLS1 gene and lipofectamine^®^ RNAiMAX transfection reagent. The siRNA target sequence was 5′-GGACAAUCCCGAAUAUGU Utt-3’. Silencer^®^ Select Negative Control No.2 siRNA (Life Technologies), which has been tested using microarray analysis to have minimal effect on gene expression profile, was used in the mock transfection as a control. 30 nM of siRNA-lipofectamine complexes were added drop-wise to the hCMEC/D3 cells. The siRNA lipofectamine complexes were then formed by mixing siRNA and RNAiMAX transfection reagents with OptiMEM in separate tubes and the two mixtures were then combined and incubated at room temperature for 5 min (as recommended by the optimized protocol for RNAiMAX transfection reagent). Cells were then incubated with these complexes overnight and the medium was changed with fresh culture medium the next day.

### mRNA expression analysis

Growing HCEMC/D3 cells were transfected with hCLS1 siRNA for 48 h. After transfection cells were harvested and total RNA obtained using the RNeasy^®^ Plus Mini kit and Qiashredder columns (Qiagen). Integrity of total RNA was confirmed by running the RNA sample on a denaturing agarose gel. Gene expression analysis was measured using an Eppendorf Mastercycler Ep Realplex System. Primers designed using NCBI/Primer-Blast were synthesized by Invitrogen (Ontario, Canada). Quantitative PCR analysis of human CLS, Creatine transporter (SLC6A8), P-glycoprotein (ABCB1), Breast cancer resistant protein (ABCG2), multidrug resistant proteins (ABCC1, ABCC3), and fatty acid transport protein-1 (FATP-1) were carried out with either one step qPCR using the Quantitect Probe RT PCR SYBR Green kit (Qiagen) or two steps qPCR using Platinum^®^ SYBR^®^ Green qPCR SuperMix-UDG (Invitrogen). The cDNA used for the two steps qPCR was synthesized from 2 ug of RNA with SuperScript^®^ II Reverse Transcriptase (Invitrogen) according to the manufacturer’s instructions. PCR amplification of the reference gene 18S RNA was performed for each sample to control for equal sample loading and also to allow normalization among samples as previously described (Nguyen et al., 2016). For one step PCR, the samples were heated for 15 min at 50°C for reverse transcription, which was followed by activation of HotStarTaq DNA Polymerase at 95°C for 15 min and then 40 cycles of 15 s denaturation at 95°C, 30 s annealing at the primer’s optimal annealing temperature and extension for 30 s at 72°C. All primers were tested for optimal annealing temperature and MgCl_2_ concentration. To ensure that the correct DNA segment was amplified in each reaction, the PCR product was either run on an agarose gel to make sure only one single band is seen at the predicted MW or melting curve was analyzed to have one peak melting temperature. For the PCR of CLS and Creatine transporter, standard curves were run to ensure that the PCR amplification efficiency of the target genes and the reference gene were similar. The PCR results were analyzed using the comparative Ct method. The relative gene expression, ^ΔΔ^Ct values, was determined by taking the difference between ^Δ^Ct sample and ^Δ^Ct Control. The 18S-RNA reference gene was normalized to 1 and each target gene was normalized and represented as the amount of gene expression remaining. All primer sequences, optimal PCR conditions and amplicon size can be found in Table 1.

### Radiolabeling of cells

Incorporation of radiolabeled creatine (as a measure of creatine transporter dependent solute transport) and uptake of radiolabeled oleate (as a measure of fatty acid transport) into cells was performed. Control and hCLS1 siRNA transfected cells were incubated with 0.1 mM (1–^14^C) oleate (bound to albumin, 1:1 molar ratio) for up to 30 min or with 9.1 nmol/well (^14^C) creatine (0.5 μCi/well) for up to 60 min and radioactivity incorporated into cells determined as described (Nguyen et al., 2016).

### Mitochondrial isolation, western blot analysis, and determination of CL levels

Mitochondrial fractions were obtained using the Mitochondrial Isolation Kit For Profiling Cultured Cells (Sigma-Aldrich) according to the manufacturer’s instructions. All isolation procedures were performed at 4°C. Control and hCLS1 siRNA transfected cells were washed with ice cold PBS, scraped and transferred into test tubes, centrifuged at 600 ×g for 5 min and the PBS removed. Cells were then incubated in 1 mL of 1x Extraction Buffer A for 15 min followed by homogenization by 50–60 strokes of a tight fitting Dounce A homogenizer to damage at least 50% of the cells. Cellular damage was checked by staining an aliquot with trypan blue and observed under a phase contrast microscope. The homogenate was centrifuged at 600 ×g for 10 min to remove cellular debris. The supernatant was then transferred to a new tube and centrifuged at 11,000 ×g for 10 min. The resulting pellet was designated the mitochondrial fraction and was re-suspended in CelLytic M Cell lysis reagent for downstream Western blot. Equal amounts of mitochondrial proteins (40 μg/lane) were loaded and separated by electrophoresis on a 12% SDS-PAGE gel. The proteins were then transferred onto a PVDF transfer membrane (Immobilon, Millipore, Bedford, MA). The presence of transferred proteins on the membrane was confirmed by staining with Ponceau S (Sigma). Membranes were blocked for 2 h at room temperature with 5% non-fat milk in 0.1% tween-20/TBS (TBS-T). Then, membranes were incubated overnight at 4°C in blocking buffers with rabbit primary antibodies against Crt1 (1:150 dilution) or cyclophilin. Expression of cyclophilin was used as the loading control. After several washes with TBS-T, membrane was incubated with HRP-linked monkey anti-rabbit secondary antibody (1:5,000) at room temperature for 1 h. Protein bands in the membranes were then visualized by enhanced chemiluminescence. The relative intensities of the bands were analyzed by ImageJ software and normalized to cyclophilin.

Lipids were extracted from cells and CL was separated using thin layer chromatography as previously described (Nguyen et al., 2016). The plates were stained with iodine and spots corresponding to CL removed and phospholipids determined as described (Rouser et al., 1970).

### Relative contribution of glycolysis and oxidative phosphorylation to the basal ATP production rate determination

The oligomycin sensitive oxygen consumption rate (OCR) and the glycolytic proton production rate (both measured under a saturating extracellular glucose concentration) were determined using a Seahorse XF24 analyzer with reagent kits as per the manufacturer’s instructions and converted to ATP production rate. The proton production rate expressed as pmol H+/min was automatically converted by the Seahorse XF-24 analyzer from the extracellular acidification rate (mpH/min) using the buffer capacity of the media and the chamber volume. The oligomycin sensitive OCR was converted to ATP production using a P/O ratio of 2.3, while the glycolytic PPR was converted to ATP production using a 1: 1 ratio, based on the fact that in glycolysis one ATP is made per lactate/proton produced (Brand, 2005).

### Statistical analysis

All data were expressed as mean ± S.D. The differences between the experimental and the control groups were evaluated by Student’s t-test. All values with *p* < 0.05 were considered statistically significant.

## Results

**FIGURE 1:**
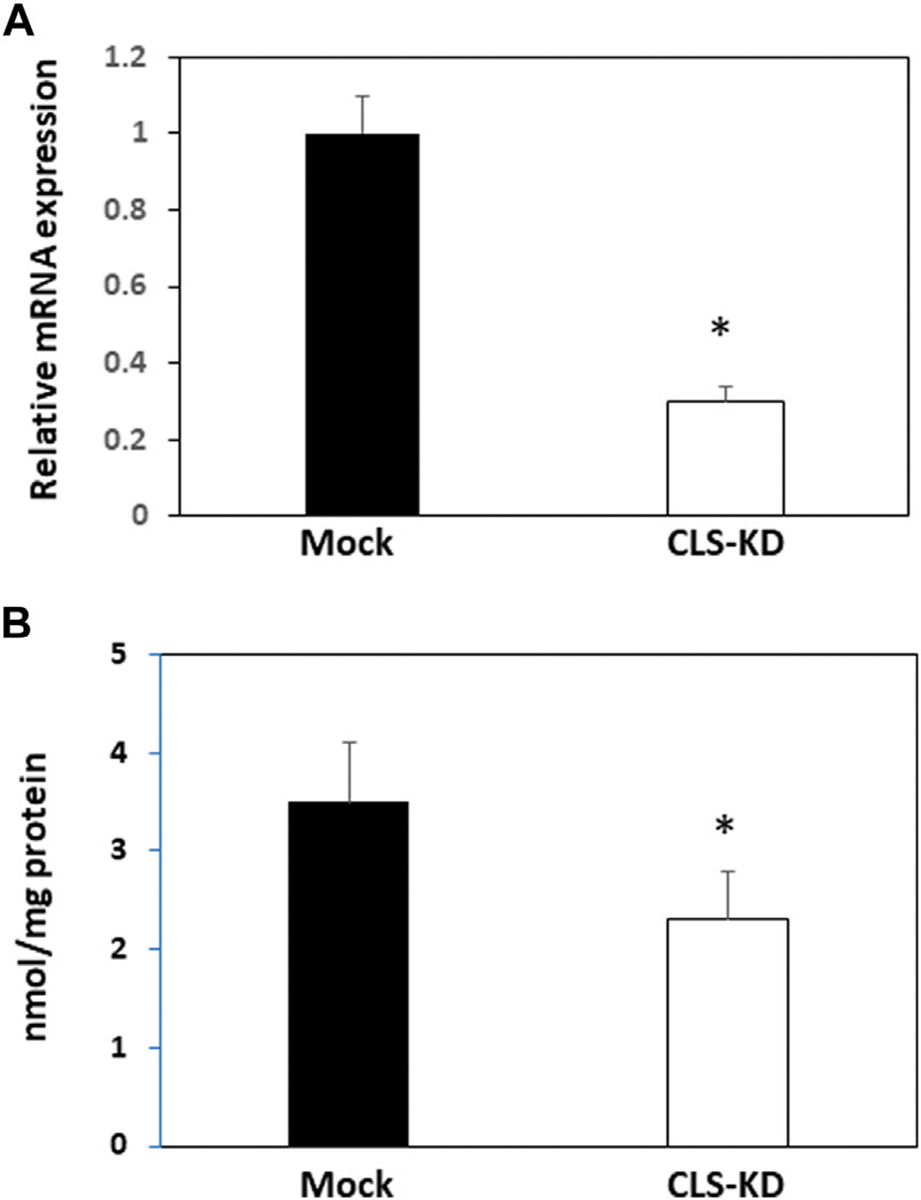
hCLS1 mRNA expression and CL levels in hCMEC/D3 cells transfected with hCLS1 siRNA. hCMEC/D3 cells were mock transfected or transfected with hCLS1 siRNA for 48 h and hCLS1 mRNA expression **(A)** or CL content **(B)** determined as described in Materials and Methods. *n* = 3–4, *p < 0.05.

Transfection of hCMEC/D3 cells with human cardiolipin synthase-1 (hCLS1) siRNA reduced hCLS1 levels approximately 70% compared to mock-transfected control cells (Figure 1A). CL levels were reduced 35% (*p* < 0.05) in hCLS1 siRNA transfected cells compared to mock-transfected controls, respectively (Figure 1B). Thus, knockdown of hCLS1 in hCMEC/D3 cells reduces CL levels.

**FIGURE 2:**
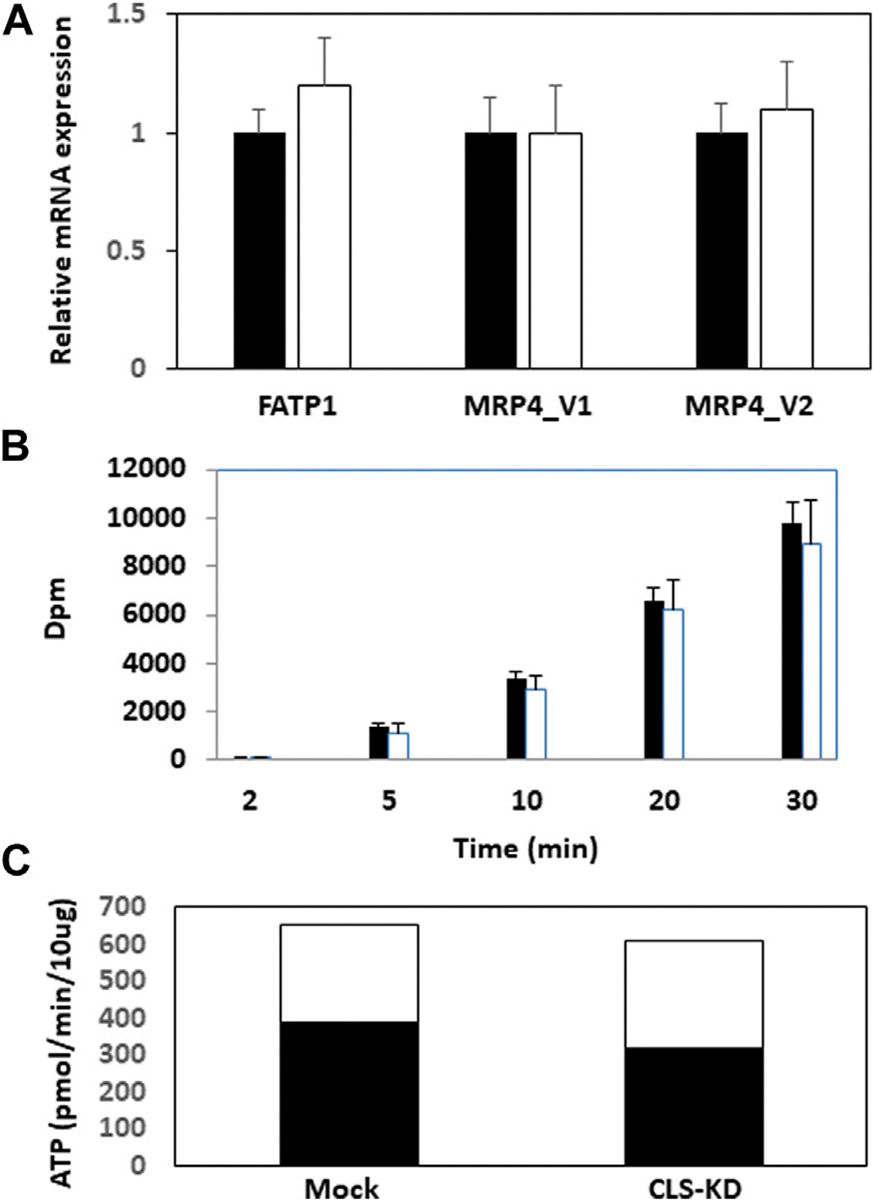
Expression of FATP1, MRP4_v1, and MRP4_v2, (1–^14^C)oleate uptake and relative contribution of oxidative phosphorylation and glycolysis to ATP production in hCMEC/D3 cells transfected with hCLS1 siRNA. hCMEC/D3 cells were mock transfected (closed bars) or transfected with hCLS1 siRNA (open bars) for 48 h and FATP1, MRP4_v1, and MRP4_v2 mRNA expression **(A),** and (1–^14^C)oleate uptake **(B)** determined. **(C)**. Relative contribution of oxidative phosphorylation (closed bars) and glycolysis (open bars) to ATP production was determined as described in Materials and Methods. *n* = 3–4, *p < 0.05.

We initially examined mRNA expression of the fatty acid transport protein FATP1 and the multidrug resistance-associated protein-4 variants MRP4_v1 and MRP4_v2 in hCMEC/D3 CLS-KD cells. mRNA expression of FATP1, MRP4_v1, and MRP4_v2 were unaltered in hCLS1 siRNA transfected cells compared to mock-transfected controls (Figure 2A). We previously determined that membrane integrity was maintained in hCMEC/D3 cells with hCLS1 siRNA knockdown (Nguyen et al., 2016). This was confirmed by examining the incorporation of (1–^14^C) oleate into hCMEC/D3 cells. Incorporation of (1–^14^C)oleate into hCMEC/D3 cells was identical between mock-transfected controls and hCLS1 siRNA transfected cells and corresponded to the lack of alteration in FATP1 mRNA expression in these cells (Figures 2A, B). Membrane integrity can be potentially compromised by loss of ATP. We confirmed that hCLS1 knock down did not affect cellular ATP production rate (Figure 2C; Supplementary Figure S1). Control hCMEC/D3 cells appeared to make ~60% of their ATP *via* OX-PHOS whereas hCLS1 knock down cells exhibited a reduced production of ATP from OX-PHOS.

**FIGURE 3:**
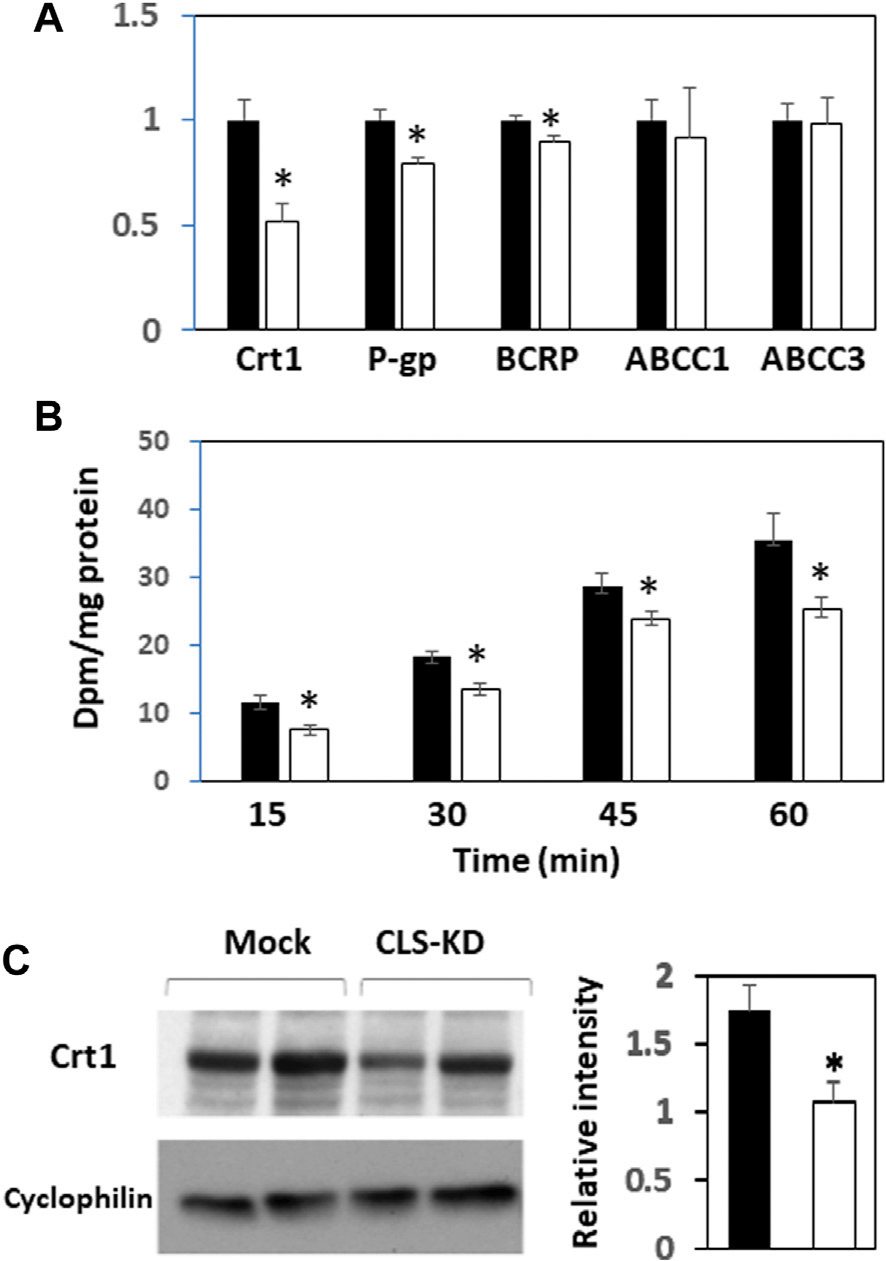
Expression of Crt1, P-gp, BCRP, ABCC1, and ABCC3, and (^14^C) creatine uptake, and Crt1 protein expression in hCMEC/D3 cells transfected with hCLS1 siRNA. hCMEC/D3 cells were mock transfected (closed bars) or transfected with hCLS1 siRNA (open bars) for 48 h and Crt1, P-gp, BCRP, ABCC1, and ABCC3 mRNA expression **(A)**, (^14^C)creatine uptake **(B)** and Crt1 protein expression **(C)** determined as described in Materials and Methods. *n* = 3–4, *p < 0.05.

We next examined mRNA expression of other major transporters in hCMEC/D3 CLS-KD cells that are expected to be found in brain endothelial cells (Dalvi et al., 2014). mRNA expression of Crt1, P-gp, and BCRP were reduced by hCLS1 knockdown but not the mRNA expression of ABCC1 and ABCC3 (Figure 3A). The greatest reduction was in Crt1 (45% decrease, *p* < 0.05). In addition, protein expression of Crt1 and (^14^C)creatine uptake into hCMEC/D3 cells were reduced by hCLS1 knockdown (Figures 3B, C). Thus, knockdown of hCLS1 in hCMEC/D3, with a corresponding reduction in CL, results in reduction in Crt1 expression and (^14^C)creatine uptake into hCMEC/D3 cells

## Discussion

In the current study we examined if deficiency in BBB endothelial CL in hCMEC/D3 cells regulate the expression of selected drug and metabolite transporters. We observed that knockdown of hCLS1 in hCMEC/D3 cells reduced CL levels and the mRNA expression of Crt1, P-gp and BCRP, but not other transporters examined. The reduction in Crt1 mRNA expression resulted in a corresponding reduction in Crt1 protein expression and a lowered incorporation of (^14^C) creatine into hCMEC/D3 cells. These data suggest that optimal CL levels are required for the regulation of creatine uptake into hCMEC/D3 cells.

Three families of ABC transporters have been characterized to play important role in the transport of therapeutic drugs across the BBB including P-glycoprotein (P-gp), the breast cancer resistance protein (BCRP), and the multi-drug resistance associated proteins (MRPs) (Loscher and Potschka, 2005). P-gp is localized on the abluminal surface of BBB endothelial cells and functions in preventing many lipophilic substances from entering into the brain. A wide range of pharmaceutical agents with molecular weights ranging from 3,000–4,000 Da are reported to be substrates of this transporter. These compounds include antibiotics, immunosuppressive agents and anti-cancer agents (Loscher and Potschka, 2005; Miller et al., 2008). P-gp expression was reported to be increased in neurological conditions such as brain tumors and epilepsy and may partly be responsible for the multi-drug resistance observed in these pathological conditions. Thus, strategies to inhibit P-gp or downregulate its expression would be advantageous in treatment of these neurological disorders. Indeed, many P-gp inhibitors have been developed and are in different stages of clinical trials (Loscher and Potschka, 2005). However, most inhibitors have failed to be clinically successful. This may, in part, be due to the fact that sufficient expression of P-gp is required under physiological conditions to keep a wide range of neurotoxins from accumulating in the brain. Several studies have demonstrated that normally well tolerated drugs become neurotoxic in the absence of P-gp due to high accumulation in the brain [reviewed in (Schinkel, 1999)]. BCRP was initially discovered in a multi-drug resistant breast cancer cell line that exhibited reduction in accumulation of chemotherapeutic drugs, even in the absence of P-gp and MRPs (Doyle et al., 1998). Due to its sequence and structural homology, it was suggested to belong to the ABCG family, a subfamily of the ABC superfamily of transporters (Schinkel and Jonker, 2003). BCRP expression is not specific to chemotherapy resistant breast cancer cells (Doyle et al., 1998). Since high expression of P-gp mediates resistance of cancers to chemotherapeutic drugs, our findings of reduced P-gp and BCRP mRNA expression in hCLS1 knockdown hCMEC/D3 cells indicate that lowering CL levels in BBB endothelial cells could potentially be used as a therapeutic approach to enhance drug entry into the brain to treat brain tumors.

MRPs are members of the ABC superfamily known to play an important role in multidrug resistance. MRP family members transport mainly anionic drugs such as methotrexate but their substrates may also include neutral agents such as glutathione and its derivatives (Borst et al., 2000). MRPs substrate specificity may overlap with P-gp to further enhance drug resistance. We observed that, in addition to FATP1, reduction in CL in hCMEC/D3 cells did not affect mRNA expression of the multidrug resistance-associated protein-4 variants MRP4_v1 and MRP4_v2 or ABCC1 and ABCC3 indicating that loss of CL impact only selected transporters in hCMEC/D3 cells.

In organs that require a high burst of energy supply during activation such as the muscle and brain, ATP is often stored in the form of phosphocreatine that can serve as an immediate substrate for ATP regeneration - a process 12 times faster than oxidative phosphorylation (OX-PHOS) (Rae et al., 2003). This is supported by the observation that phosphocreatine levels decrease rapidly during brain activation while ATP levels remain constant. Whether creatine is primarily synthesized in brain or is imported from the peripheral circulation remains a subject of debate. Crt1 is encoded by the SLC6A8 gene and exhibits the ability to uptake creatine into the brain (Ohtsuki et al., 2002; Beard and Braissant, 2010). However, as expression of this transporter is absent in astrocytic end feet covering the endothelium, it was suggested that permeability of creatine into the brain might be limited (Beard and Braissant, 2010). This may be true as oral administration of 20 g of creatine per day consecutively for 4 weeks in healthy volunteers resulted in only a moderate 5%–10% increase in brain creatine levels (Dechent et al., 1999). Since the brain is also known to express both L-arginine: glycine amidinotransferase and guanidinoacetate methyltransferase, enzymes required for endogenous creatine synthesis, it has been suggested that the brain can self-supply most of its required creatine (Beard and Braissant, 2010). This is supported by the observation that treatment of L-arginine:glycine amidinotransferase and guanidinoacetate methyltransferase deficient patients with a high oral doses of creatine partially replenished cerebral creatine levels but did not impact these patients central nervous system developmental alterations (Braissant, 2012). These observations challenge the importance of Crt1 in supplying creatine to the brain. However, the fact that patients with defects in the SLC6A8 gene experience progressive brain atrophy, cognitive disability and speech deficit, which cannot be treated by creatine oral supplementation, indicate an essential role of Crt1 in maintaining optimal brain creatine levels. In the current study, the reduction in uptake of creatine was not likely due to reduction in cellular ATP due to reduced CL since ATP production rate appeared to be maintained in cells with knockdown of hCLS1. This is in agreement with our previous study in which CLS knockdown with reduced CL resulted in an increased glucose uptake and glycolysis in order to maintain cellular ATP levels (Nguyen et al., 2016). Interestingly, control hCMEC/D3 cells appeared to make ~60% of their ATP *via* OX-PHOS under basal condition. This supports the hypothesis that, unlike endothelial cells in non-BBB regions, brain capillary endothelial cells may be more dependent on OX-PHOS for their energy requirements under basal conditions.

A limitation of our study is that it does not address whether the reduction in CL plays a direct or indirect role on membrane transport. The mechanism of this will be determined in a future study. In addition, the high passage number and transformed nature of these cells may potentially influence cell marker expression and functionality. However, the observation that FATP1 mRNA expression and (1–^14^C) oleate uptake, a process dependent on energy, are unaltered in hCMEC/D3 cells with knockdown of CLS coupled with a normal ATP production rate in these cells indicate that the effect of reduced CL on (^14^C) creatine transport is not simply due to alteration in energy metabolism.

## Supplementary Material

Suppl. Fig. 1

## Figures and Tables

**TABLE 1:** Primers and annealing temperatures for RT-PCR and amplicon size.

Primer	Forward 5’–3’	Reverse 5’–3’	Annealing °C	Size
hCLS1	AATGACGAGAATTGGCTGG	TCTTTGATTGGCCCAGTTTC	60	142 bp
FATP1	ACTCGGCAGGAAACATCATC	TCTCCCCGATGTACTGAACC	60	141 bp
Crt1	AGTCCTTTACCACCACGCTG	GACAAAGGGTCACCTCCCAG	60	234 bp
MRP4_V1	CCATCTGTGCCATGTTTGTC	ACTGAAACATCCCCATGAGC	60	123 bp
MRP4_V2	CCATCTGTGCCATGTTTGTC	ACTGAAACATCCCCATGAGC	60	123 bp
BCRP	GGCCTTGGGATACTTTGAATC	GAATCTCCATTAATGATGTCCA	60	90 bp
P-gp	TGCTCAGACAGGATGTGAGTTG	ATTACAGCAAGCCTGGAACC	62–63	121 bp
ABCC1	ACGCCCTTTCTGGTGGCCTT	TTGACAGGCCGTCGCTCGAT	62	242 bp
ABCC3	CGCGCCTTCCAGGTAAAGCAAA	TGTGCCAAGCCTCACCAGGA	60	231 bp

## Data Availability

The original contributions presented in the study are included in the article/Supplementary Material, further inquiries can be directed to the corresponding author.
