# Supplementary figures and images for: Reduction in cardiolipin reduces expression of creatine transporter-1 and creatine transport in growing hCMEC/D3 human brain microvessel endothelial cells
Source: Front Drug Deliv. 2023 Mar 29;3:1158369. doi: 10.3389/fddev.2023.1158369 (PMC11906176; doi:10.3389/fddev.2023.1158369)

**Supplementary Figure 1**

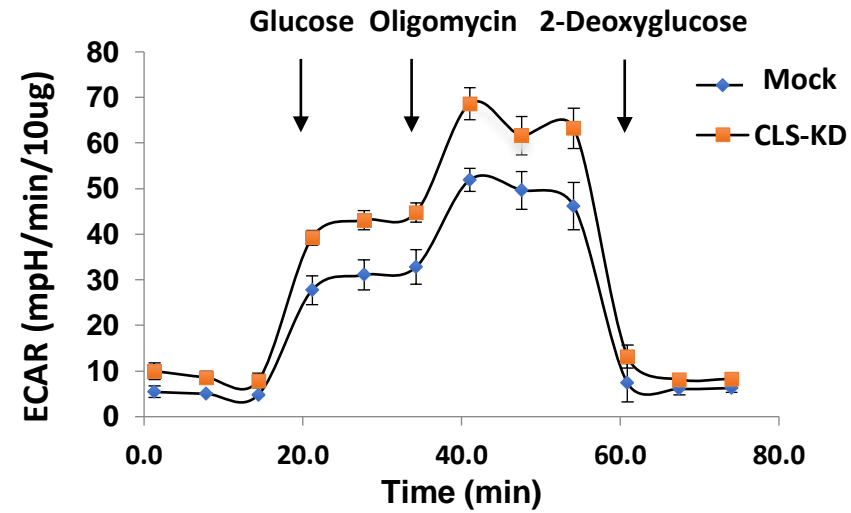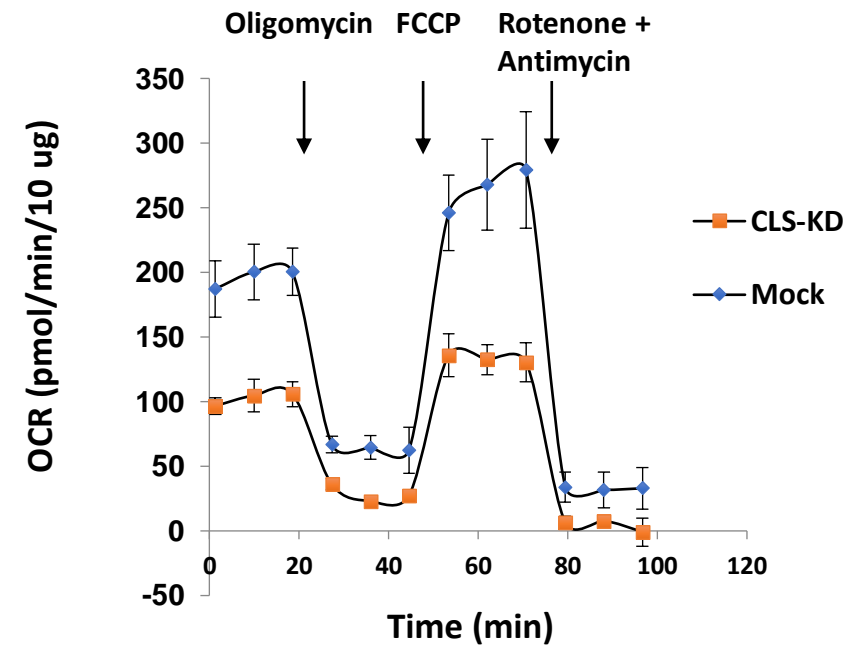

Supplement: Supplementary file 1 [file DataSheet1.PDF]
